# Effects of long-chain fatty acid supplementation on the growth performance of grower and finisher pigs: a meta-analysis

**DOI:** 10.1186/s40104-019-0374-1

**Published:** 2019-08-16

**Authors:** Zhi Li, Bocheng Xu, Zeqing Lu, Yizhen Wang

**Affiliations:** National Engineering Laboratory of Biological Feed Safety and Pollution Prevention and Control, Key Laboratory of Animal Nutrition and Feed of Ministry of Agriculture, Key Laboratory of Animal Nutrition and Feed Science of Zhejiang Province, Institute of Feed Science, Zhejiang University, 866 Yuhangtang Road, Hangzhou, Zhejiang Province 310058 People’s Republic of China

**Keywords:** Energy density, Finisher pig, Grower pig, Growth performance, Long-chain fatty acid, Meta-analysis, Production performance

## Abstract

**Background:**

Supplementation of feed with long-chain fatty acids (LCFAs) during the grower and finisher phases has long been discussed as a growth promotion strategy in pigs, but its effects are inconsistent. The purpose of our study was to comprehensively evaluate its effects on the growth performance based on the average daily gain (ADG), average daily feed intake (ADFI) and gain: feed (G:F) ratio and to unveil the roles of the basal diet, LCFA concentration and LCFA saturation.

**Results:**

We searched the PubMed and Web of Science databases (articles published from Jan 1^st^, 2000, to Sep 30^th^, 2018; restricted to English) and compared LCFA-supplemented diets with control diets. We retrieved 2346 studies, 18 of which (1314 pigs, 26 records) were eligible for our analysis. We used a random-effects model to calculate the weighted mean differences (WMDs) and 95% confidence intervals (CIs). LCFA supplementation in the grower-finisher phase improved the ADG (WMD = 41.74 g/d, 95% CI: 8.81 to 74.66, *P* = 0.013) and G:F ratio (WMD = 0.019, 95% CI: 0.006 to 0.032, *P* = 0.003). For supplementation solely in the finisher phase, we found a similar performance in the ADG (WMD = 39.93 g/d, 95% CI: 26.48 to 53.38, *P* < 0.001) and G:F ratio (WMD = 0.019, 95% CI: 0.006 to 0.032, *P* < 0.001) but a reduction in the ADFI (WMD = − 83.863 g/d, 95% CI: − 156.157 to − 11.569, *P* = 0.023). Specifically, approximately 5% LCFA supplementation in the finisher phase had significant effects on the ADG (WMD = 51.385 g/d, 95% CI: 35.816 to 66.954, *P* < 0.001), ADFI (WMD = − 102.869 g/d, 95% CI: − 189.236 to − 16.502, *P* = 0.02) and G:F ratio (WMD = 0.028, 95% CI: 0.018 to 0.039, *P* < 0.001), whereas a concentration of approximately 1% exhibited no effects.

**Conclusions:**

Overall, regardless of the basal diet and saturation, LCFA supplementation greatly improves the growth performance of grower and finisher pigs, primarily by increasing the energy density.

## Background

Antimicrobial growth promoters (AGPs) have been used in the pig industry for more than 60 years, where they have made impressive contributions in terms of economic benefits and healthy farming [1]. Simultaneously, as a result of extensive use of sub-therapeutic antimicrobials [2], pigs have become an important reservoir of antimicrobial-resistant bacterial strains and genes, which seriously endanger public health [3, 4]. In addition, rapidly rising concerns about food safety from consumers are encouraging animal nutritionists to develop reliable alternatives for growth promotion.

Supplementation of long-chain fatty acids (LCFAs), which is the largest category of fatty acids in animal diets [5], or their compounds into daily rations provides a practical method for achieving better growth performance than diets without additional LCFA supplementation. More importantly, few adverse effects of fatty acids have been found, which ensures easier acceptance of LCFAs as a growth promoter by consumers. According to the National Research Council (NRC) [6], supplementation of grower and finisher feed with fatty acids increases the growth speed, reduces the feed intake and improves the gain efficiency. However, in feeding trials, the effects of LCFA supplementation on growth performance are inconsistent. Our review of previous studies reveals that some influential factors, including the basal diet [corn-soybean vs. distillers’ dried grains with solubles (DDGS)], LCFA concentration (high concentration vs. low concentration) and LCFA saturation (saturated vs. unsaturated), should be considered when exploring the synergistic effects of basal diet with LCFAs, the dosage-dependent effect of such supplementation and the influence of the physicochemical properties of LCFAs, respectively.

Because separate studies differ in the factors considered and inevitably lack an overall investigation [7], we performed a meta-analysis to reveal the effects of LCFA supplementation on grower and finisher pigs and to elucidate the influential factors based on the average daily gain (ADG), average daily feed intake (ADFI) and gain:feed (G:F) ratio.

## Methods

This meta-analysis strictly followed the guidelines of the Preferred Reporting Items for Systematic Reviews and Meta-analyses [8].

### Search strategy

We collected relevant studies published between Jan 1^st^, 2000, and Sep 30^th^, 2018, in the PubMed (https://www.ncbi.nlm.nih.gov/pubmed; accessed Sep 30^th^, 2018) and Web of Science (http://webofknowledge.com; accessed Sep 30^th^, 2018) databases. The date range was chosen based on the development of feeding facilities and improvements in growth performance due to breeding [9]. We restricted the language to English. The search principles were as follows: 1) the terms “grower pig” and “finisher pig” were extended to include “pig”, “swine”, “boar”, “piglet”, “sow”, “gilt” and “barrow”; 2) the terms related to fatty acids were searched in the PubMed database beforehand and shown to be “acids, fatty”, “fatty acids, esterified”, “acids, esterified fatty”, “esterified fatty acids”, “fatty acids, saturated”, “acids, saturated fatty”, “saturated fatty acids”, “aliphatic acids” and “acids, aliphatic”; and 3) growth performance was equal to production performance. The detailed search strategy and findings are shown in Table 1. We also extended our search to the articles referenced by the studies identified for the meta-analysis.

**Table 1:** Search strategy

Search	Query	Items found
PubMed		
#1	Search: (((((pig) OR swine) OR boar) OR piglet) OR gilt) sow) OR barrow)); Filters: Publication date from 2000/01/01 to 2018/09/30	138,595
#2	Search: ((((((((((fatty acid) OR Acids, Fatty) OR Fatty Acids, Esterified) OR Acids, Esterified Fatty) OR Esterified Fatty Acids) OR Fatty Acids, Saturated) OR Acids, Saturated Fatty) OR Saturated Fatty Acids) OR Aliphatic Acids) OR Acids, Aliphatic)); Filters: Publication date from 2000/01/01 to 2018/09/30	278,866
#3	Search: (growth performance) or (production performance); Filters: Publication date from 2000/01/01 to 2018/09/30	88,993
#1 AND #2 AND #3		377
Web of Science		
#1	TS = (pig OR piglet OR sow OR gilt OR barrow OR boar OR swine)	453,157
#2	TS = (fatty acid OR Acids, Fatty OR Fatty Acids, Esterified OR Acids, Esterified Fatty OR Esterified Fatty Acids OR Fatty Acids, Saturated OR Acids, Saturated Fatty OR Saturated Fatty Acids OR Aliphatic Acids OR Acids, Aliphatic)	489,479
#3	TS = (growth performance OR production performance)	2,068
#1 AND #2 AND #3		1,969

### Selection criteria and procedure

**Fig. 1:**
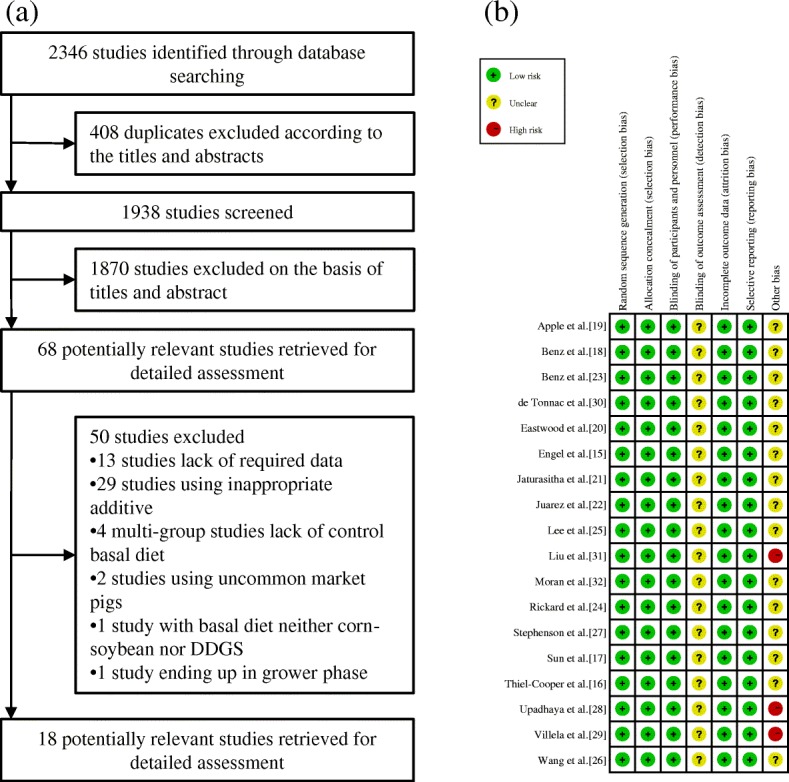
Study selection process and quality assessment. **a** Study selection process. **b** Study quality assessment

Studies were eligible for inclusion in our meta-analysis under the conditions that 1) the growth performance parameters (ADG, ADFI and G:F ratio) were reported, 2) LCFAs, LCFA esters, LCFA-rich compounds or LCFA salts were added to the feed throughout the experimental period, 3) the trials were initiated at the growing or finishing phase and terminated at the finishing phase, 4) the ADFI and G:F ratio were calculated by the gross weight of the feed, 5) the genetic background was a commercial breed (e.g., Duroc × Landrace × Yorkshire) and 6) the detailed fatty acid composition and protein density were included, with no difference in the protein level. The exclusion criteria were as follows: 1) the major content of the supplement was not LCFAs (e.g., grape seed cake and rice bran); 2) the study lacked a controlled diet without additional fatty acid supplementation; and 3) the basal diet was not corn-soybean or DDGS (e.g., barley diet). Based on these standards, we selectively screened eligible studies for inclusion in the analysis (Fig. 1a).

The information extracted from the included studies was as follows: author information (first author, year); genetic background; sum number of pigs included in the control and treatment groups; mean initial body weight; mean final body weight; initial phase (grower or finisher); supplemental substance; energy difference; basal diet (corn-soybean or DDGS); concentration (low or high); saturation (saturated or unsaturated); and growth performance (ADG, ADFI and G:F ratio) of the treatment and control groups. One study could have more than one record depending on the treatments and growth phases of the pigs.

The study selection procedure was as follows: 1) two investigators (Z. Li and B. Xu) independently screened the titles and abstracts of the acquired studies and identified relevant studies for full-text reading; 2) disagreements during independent study selection were referred to the third investigator (Y. Wang) for an ultimate resolution; and 3) after the eligible studies were verified according to our criteria, one investigator (Z. Li) extracted the data and information from each study, followed by inspection by the other investigator (B. Xu).

### Study quality assessment

Two investigators (Z. Li and B. Xu) performed independent study quality assessment according to the criteria provided in the Consolidated Standards of Reporting Trials statement [8] and the Cochrane Collaboration’s tool for assessing risk of bias [10]. The assessment aspects included sequence generation, allocation concealment, blinding of participants and personnel, incomplete outcome data, selective reporting and other bias. The divergences were resolved by the third investigator (Y. Wang) (Fig. 1b).

### Within-group SD estimate

Within-group standard deviations (SDs) or standard errors (SEs) are required for a meta-analysis. However, in the included articles, these data usually were missing, and the provided SE of the mean could not be used to calculate the within-group SD. In such cases, first we contacted the authors via email to request the within-group SDs. If raw statistics were not available from the authors, the within-group SDs of growth performance were estimated using 8–15% of the mean value, which was based on the raw statistics of our institute, suggestions from peers in both industry and college settings and relevant data presented by the NRC [6]. Technically, the SD is derived from the random errors in trials and follows a random distribution. Thus, the SD should not be calculated as a single mean ratio. To be prudent, we randomized the SDs of each group and repeated the meta-analysis 10 times to confirm the stability of our results. If we had observed a statistically significant difference, then the meta-analysis would have been regarded as impossible for this topic and ceased. The data used for the subsequent analyses and presentation originated from one of the 10 random processes.

### Statistical analysis

The statistical analysis was performed with Stata 12.0 (Stata Corp., USA).

### Meta-analysis

We calculated the pooled estimates of the mean differences between the treatment and control groups using a random-effects model [11]. We also used Cochran’s Q statistic (significance level of *P* ≤ 0.1) and the *I*^2^ statistic to quantitatively measure the heterogeneity in our analysis. The grading of heterogeneity was as follows: no heterogeneity, *I*^2^ ≤ 25%; low heterogeneity, 25% < *I*^2^ ≤ 50%; moderate heterogeneity, 50% < *I*^2^ ≤ 75%; and high heterogeneity, *I*^2^ > 75% [12].

### Regression analysis

To measure the effects of covariants, which in our study are the basal diet, LCFA concentration and LCFA saturation, on the outcomes (ADG, ADFI and G: F ratio), we performed a regression analysis after the meta-analysis. To avoid a false positive result, the regression analysis was applied only to groups with more than 10 records.

### Subgroup categorization and analysis

We conducted subgroup analyses to elucidate heterogeneity that was significant (*P* < 0.05) or beyond a moderate level (*I*^2^ > 50%). The included studies were classified into the “corn-soybean diet vs DDGS diet”, “high concentration vs low concentration” and “saturated vs unsaturated” subgroups. The low and high concentrations were set to approximately 1% and 5%, respectively, based on the frequency of occurrence in the included studies and, more importantly, the role of fatty acids in the feed. Low-concentration supplementation (approximately 1%) often applied to unsaturated fatty acids (e.g., conjugated linoleic acid) and was more likely to have biological functions, including biomembrane constitution, signal transduction [13, 14] and eicosanoid precursor action (e.g., prostaglandins, leukotrienes and thromboxanes) [15]. The saturation classification of fatty acid compounds (e.g., animal fat and vegetable oils) was determined by the dominating ratio (> 50%) in the fatty acid composition [16].

### Sensitivity analysis

If the heterogeneity was significant (*P* < 0.05), a sensitivity analysis was performed to identify the study (or studies) that contributed as the main source of the heterogeneity. Heterogeneity and pooled estimates were recalculated after the study or studies (including all records) was deleted from the outcome.

### Publication bias

Publication bias was evaluated using Begg’s and Egger’s tests, for which the significance level was defined at *P* < 0.1 [11]. If Begg’s and Egger’s tests disagreed, Egger’s test was used as a reference. Additionally, the trim-and-fill test was used to further test and adjust for publication bias [17].

## Results

Of the 2346 studies identified, we included 18 studies (with data for 1314 pigs) and extracted 26 records for our meta-analysis [18–35]. Except for Juarez et al. [25], in which the pigs were from a commercial farm, all of the studies were performed on clearly defined commercial breeds, with 5 studies (6 records) beginning LCFA supplementation at the grower phase and 13 studies (20 records) beginning it at the finisher phase. The mean initial weights of the growing and finishing pigs were 33.98 kg and 67.78 kg, respectively. All studies ended in the finishing phase, with a mean body weight of 115.32 kg. The categorization of growth phases was combined with both the body weights and the experimental design (Table 2). Because of the nonsignificant publication bias (*P* > 0.1) in the current meta-analysis, the trim-and-fill test was not performed (Table 3).

**Table 2:** Characteristics of the included studies

Study	Year	Genetic background	*N* ^a^	Initial BW^b^, kg	Final BW^b^, kg	Initial phase	Supplemental substance^c^	Energy difference^d^	Basal diet	Concentration	Saturation	ADG, g/d^e^		ADFI, g/d^e^		G:F ratio^e^	
												T	CT	T	CT	T	CT
Engel et al. [18]	2001	PIC L326 boars × C15 sows	36	60	110	Finisher	Choice white grease	→	Corn-soybean	High (4%)	Unsaturated	980	920	3140	3130	0.3	0.31
				60	110	Finisher	Poultry fat	→	Corn-soybean	High (4%)	Unsaturated	930	920	3140	3130	0.31	0.31
Thiel-Cooper et al. [19]	2001	Yorkshire × Landrace × Duroc × Hampshire	16	26.3	116	Grower	Conjugated linoleic acid	N.A.	Corn-soybean	Low (1%)	Unsaturated	1019^*^	942	2634	2683	0.384^*^	0.352
Sun et al. [20]	2004	Duroc × Landrace × Yorkshire	36	63.8	98.9	Finisher	Conjugated linoleic acid	→	Corn-soybean	High (4%)	Unsaturated	890^**^	780	2700^**^	2510	0.328^**^	0.312
Benz et al. [21]	2007	TR4 × 1050	40	54.39	122.47	Finisher	Choice white grease	↑	Corn-soybean	High (5%)	Unsaturated	994.26	921.62	2651.36	2596.88	0.374	0.356
Apple et al. [22]	2008	Mating of line 348 sows to EB boars	108	77.9	108.9	Finisher	Beef tallow	↑	Corn-soybean	High (5%)	Saturated	810	780	2060	2030	0.4	0.39
Eastwood et al. [23]	2009	Camborough Plus sows × C337 boars	100	32	115	Grower	Flaxseed meal	↑	Corn-soybean	High (5%)	Unsaturated	937	948	2648	2657	0.36	0.36
Jaturasitha et al. [24]	2009	Duroc × Yorkshire × Landrace	300	35	90	Grower	Tuna oil	↑	Corn-soybean	Low (1%)	Unsaturated	707	645	2000	1990	0.354	0.324
Juarez et al. [25]	2010	Commercial pigs	16	31	84	Grower	Co-extruded flaxseed	↑	Corn-soybean	High (5%)	Unsaturated	1000	940	2600^*^	2460	0.39^*^	0.38
Benz et al. [26]	2011	327 × PIC C22	48	44	123	Finisher	Choice white grease	↑	Corn-soybean	High (5%)	Unsaturated	1040	990	2960	3150	0.38^**^	0.35
				44	123	Finisher	Soybean oil	↑	Corn-soybean	High (5%)	Unsaturated	1070^**^	990	3110	3150	0.39^**^	0.35
Rickard et al. [27]	2012	PIC 337	24	100.68	136	Finisher	Conjugated linoleic acid	↓	DDGS	Low (0.6%)	Unsaturated	1280	1240	3390	3370	0.17^**^	0.17
Lee et al. [28]	2013	Landrace × Yorkshire × Duroc	36	43.7	128.9	Finisher	Beef tallow	↑	DDGS	High (3%)	Saturated	980	950	2700	2670	0.37	0.36
				43.7	128.9	Finisher	Palm kernel oil	↑	DDGS	High (3%)	Saturated	980	950	2540	2670	0.39	0.36
Wang et al. [29]	2015	Duroc × Landrace × Large	16	60	94.2	Finisher	Conjugated linoleic acid	↑	DDGS	Low (1%)	Unsaturated	850	840	2670	2630	0.32	0.32
Stephenson et al. [30]	2016	PIC 327 × 1050	48	45.6	132.17	Grower	Beef tallow	↑	Corn-soybean	High (4%)	Saturated	1034	1002	2706	2756	0.384^*^	0.365
				45.6	132.17	Grower	Soybean oil	↑	Corn-soybean	High (4%)	Unsaturated	1056	1002	2760	2756	0.385	0.365
Upadhaya et al. [31]	2017	Yorkshire × Landrace × Duroc	60	80.82	110.3	Finisher	Conjugated linoleic acid	→	Corn-soybean	Low (1%)	Unsaturated	839	846	2593	2614	0.323	0.324
Villela et al. [32]	2017	Duroc × Yorkshire × Landrace	143	55	90	Finisher	Cotton seed oil	↑	DDGS	High (5%)	Unsaturated	1020	970	2560^*^	2810	0.4^*^	0.34
				90	120	Finisher	Cotton seed oil	↑	DDGS	High (5%)	Unsaturated	920	870	2810^*^	3050	0.33^*^	0.28
De Tonnac et al. [33]	2017	Yorkshire × Landrace × Pietrain	23	50.7	115.21	Finisher	DHA-rich algae	↑	Corn-soybean	High (4.1%)	Unsaturated	1070	1070	3300	3300	0.357	0.345
Liu et al. [34]	2018	C22 sows × PIC L337 boars	120	73	129.94	Finisher	Soybean oil	↑	Corn-soybean	High (6%)	Unsaturated	1170	1155	3221^*^	3654	0.36^*^	0.322
				73	129.94	Finisher	Choice white grease	↑	Corn-soybean	High (6%)	Unsaturated	1226	1155	3355^*^	3654	0.367^*^	0.322
				73	129.94	Finisher	Palm oil	↑	Corn-soybean	High (6%)	Saturated	1259	1155	3559	3654	0.357^*^	0.322
				73	129.94	Finisher	Beef tallow	↑	Corn-soybean	High (6%)	Saturated	1222	1155	3399^*^	3654	0.362^*^	0.322
Moran et al. [35]	2018	PIC × Goland	144	117.1	140.75	Finisher	DHA-rich microalgae	↑	Corn-soybean	Low (1%)	Unsaturated	847.7	838.3	3392	3405	0.25	0.244

^a^Number of pigs included in our analyses

^b^*BW* body weight

^c^*DHA* docosahexaenoic acid

^d^↑, higher energy density in treatment group; →, similar energy density in treatment and control groups; ↓, lower energy density in treatment group; *N.A* not applicable

^e^*T* treatment, *CT* control; a significant difference in the trial is indicated by ^*^P < 0.05 and ^**^P < 0.01

**Table 3:** Publication bias analysis of the included studies

Outcome	Initial phase	Begg’s test	Egger’s test
ADG, g/d	Grower	0.431	0.431
	Finisher	0.394	0.394
ADFI, g/d	Grower	0.934	0.934
	Finisher	0.880	0.880
G:F ratio	Grower	0.299	0.299
	Finisher	0.616	0.616

### Effects of LCFA supplementation on the growth performance of grower-finisher pigs

**Fig. 2:**
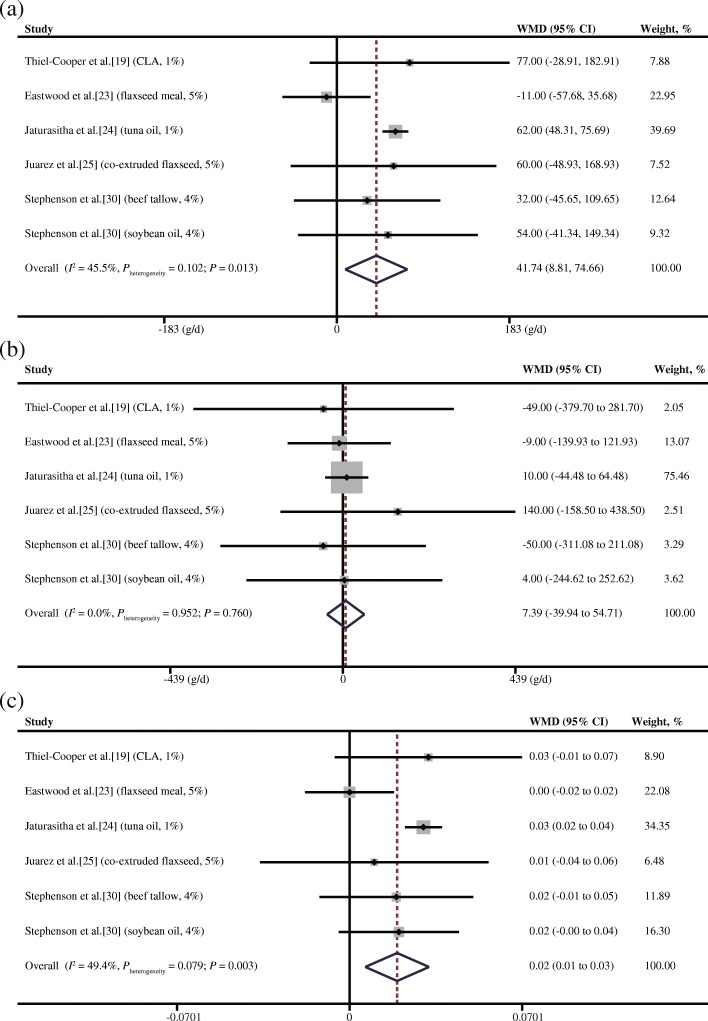
Meta-analysis of differences in the growth performances of grower pigs fed LCFAs. **a** ADG. **b** ADFI. **c** G:F ratio. CI = confidence interval; CLA = conjugated linoleic acid; CWG = choice white grease; WMD = weighted mean difference; *P*_heterogeneity_ = *P* value of heterogeneity (significance level *P*_heterogeneity_ < 0.05). The small solid diamond represents the point estimate for each individual trial, and the horizontal line extending from each solid diamond represents the upper and lower limits of the 95% CI. The size of the shaded square indicates the relative weight of the trial in the meta-analysis. Small solid diamonds located on the positive quadrant of the *X*-axis favour an increase in the growth parameters (ADG, ADFI and G:F ratio), whereas those on the negative quadrant favour a decrease. The open diamond represents the WMD and 95% CI of the trials

In Fig. 2, we present the overall effects of LCFA supplementation on the growth performance of grower-finisher pigs. Specifically, LCFA supplementation increased the ADG by 41.738 g/d (95% confidence interval (CI): 8.813 to 74.662, *P* = 0.013) with low heterogeneity (*I*^2^ = 45.5%, *P*_heterogeneity_ = 0.102) (Fig. 2a). However, LCFA supplementation had no effect on the ADFI (WMD = 7.388 g/d, 95% CI: − 39.937 to 54.713, *P* = 0.76) with no heterogeneity (*I*^2^ = 0.0%, *P*_heterogeneity_ = 0.952) (Fig. 2b). LCFA supplementation increased the G:F ratio by 0.019 (95% CI: 0.006 to 0.032, *P* = 0.003) with low heterogeneity (*I*^2^ = 49.4%, *P*_heterogeneity_ = 0.079) (Fig. 2c).

### Regression analysis

According to the regression analysis of LCFA supplementation in finisher pigs (Table 4), the LCFA concentration might play a significant role, especially in determining the ADG (*P*_regression_ = 0.014) and G:F ratio (*P*_regression_ = 0.007). In contrast, the basal diet and LCFA saturation were not major causes of heterogeneity, because they exhibited no significant effects (*P*_regression_ > 0.05) in the regression analyses of the ADG, ADFI, and G:F ratio. Therefore, we focused on the role of the concentration and conducted corresponding subgroup analyses in the subsequent research.

**Table 4:** Regression analyses of finisher pig studies included in the meta-analysis

Outcome	Subgroup	*P*_regression_^a^
ADG, g/d	Basal diet	0.773
	Concentration	0.014
	Saturation	0.710
ADFI, g/d	Basal diet	0.358
	Concentration	0.234
	Saturation	0.620
G:F ratio	Basal diet	0.107
	Concentration	0.007
	Saturation	0.724

^a^*P* value of regression, significance level *P*_regression_ < 0.05

### Effects of LCFA supplementation on the ADG of finisher pigs

**Fig. 3:**
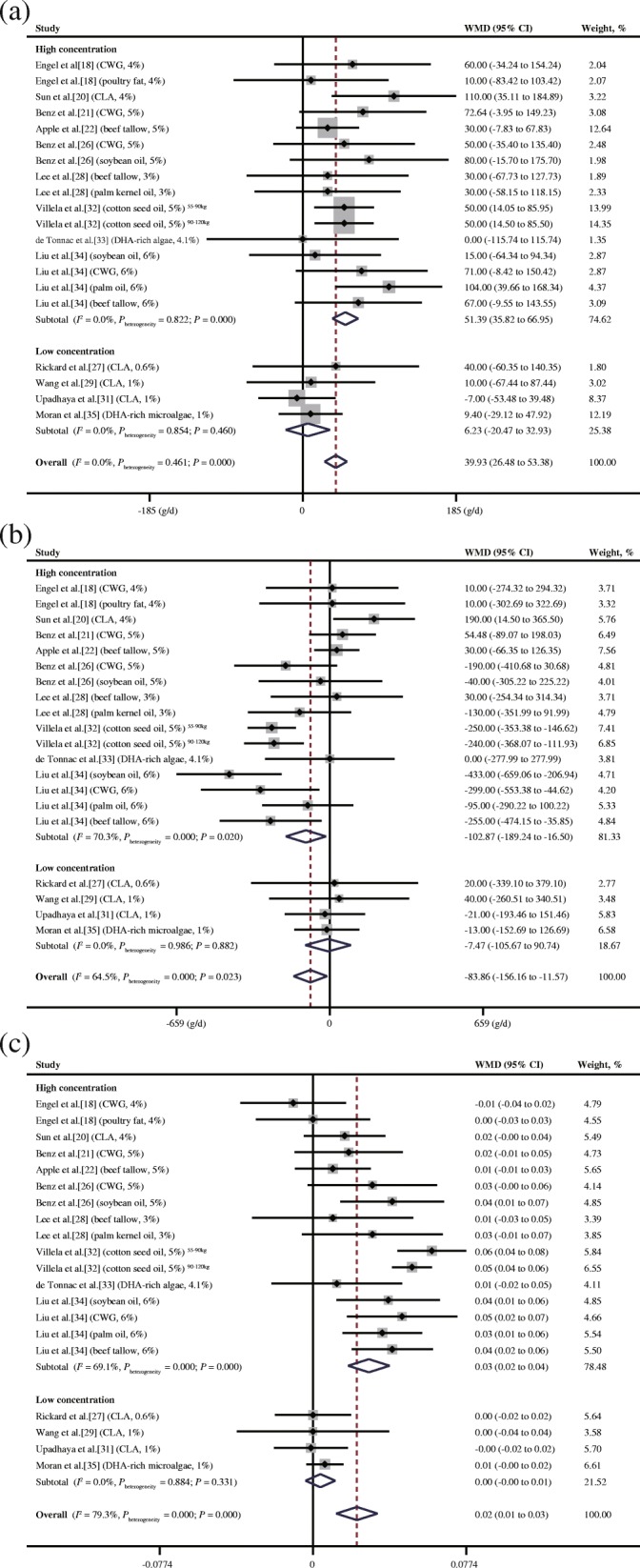
Meta-analysis of differences in the growth performances of finisher pigs fed a high/low LCFA concentration. **a** ADG. **b** ADFI. **c** G:F ratio. CI = confidence interval; CLA = conjugated linoleic acid; CWG = choice white grease; WMD = weighted mean difference; *P*_heterogeneity_ = *P* value of heterogeneity (significance level *P*_heterogeneity_ < 0.05). The small solid diamond represents the point estimate for each individual trial, and the horizontal line extending from each solid diamond represents the upper and lower limits of the 95% CI. The size of the shaded square indicates the relative weight of the trial in the meta-analysis. Small solid diamonds located on the positive quadrant of the *X*-axis favour an increase in the growth parameters (ADG, ADFI and G:F ratio), whereas those on the negative quadrant favour a decrease. The open diamond represents the WMD and 95% CI of the trials

As shown in Fig. 3a, LCFA supplementation increased the ADG by 39.926 g/d (95% CI: 26.477 to 53.375, *P* = 0.000) with no heterogeneity (*I*^2^ = 0.0%, *P*_heterogeneity_ = 0.461). Specifically, the high concentration increased the ADG by 51.385 g/d (95% CI: 35.816 to 66.954, *P* = 0.000) with no heterogeneity (*I*^2^ = 0.0%, *P*_heterogeneity_ = 0.822), whereas the low concentration did not influence the ADG (WMD = 6.227 g/d, 95% CI: − 20.471 to 32.926, *P* = 0.460) with no heterogeneity (*I*^2^ = 0.0%, *P*_heterogeneity_ = 0.854).

### Effects of LCFA supplementation on the ADFI of finisher pigs

As presented in Fig. 3b, compared with the ADFI of pigs on the basal diet, LCFA supplementation significantly decreased the ADFI (WMD = − 83.863 g/d, 95% CI: − 156.157 to − 11.569, *P* = 0.023) with moderate heterogeneity (*I*^2^ = 64.5%, *P*_heterogeneity_ = 0.023). Only the high concentration reduced the ADFI (WMD = − 102.869 g/d, 95% CI: − 189.236 to − 16.502, *P* = 0.02), whereas the low LCFA concentration had no effect (WMD = − 7.466 g/d, 95% CI: − 105.667 to 90.735, *P* = 0.882). We observed a moderate level of heterogeneity (*I*^2^ = 70.3%, *P*_heterogeneity_ = 0.000) in the high-concentration subgroup and no heterogeneity (*I*^2^ = 0.0%, *P*_heterogeneity_ = 0.986) in the low-concentration subgroup.

### Effects of LCFA supplementation on the G:F ratio of finisher pigs

As shown in Fig. 3c, LCFAs had a significant positive effect on the G:F ratio of the finishers (WMD = 0.022, 95% CI: 0.012 to 0.033, *P* = 0.000), which was especially strong in the case of high-concentration supplementation (WMD = 0.028, 95% CI: 0.018 to 0.039, *P* = 0.000). In contrast, low-concentration supplementation of finisher feed had no effect on the G:F ratio (WMD = 0.004, 95% CI: − 0.004 to 0.011, *P* = 0.331). Although high heterogeneity was detected overall (*I*^2^ = 79.3%, *P*_heterogeneity_ = 0.000), after the subgroup analysis, the majority of the heterogeneity was attributed to the high-concentration subgroup (*I*^2^ = 69.1%, *P*_heterogeneity_ = 0.000) rather than the low-concentration subgroup (*I*^2^ = 0.0%, *P*_heterogeneity_ = 0.884).

## Discussion

In swine husbandry, AGPs have been shown to be unquestionably stable and to have excellent effects on growth performance and animal health [36]. Therefore, with global banning of AGPs, a long list of alternatives (including antimicrobial peptides, organic acids, enzymes, probiotics, prebiotics, essential oils and metal oxides) has been intensively researched and developed to compensate for the vacancy [37, 38]. These alternative substances exhibit similar or even better effects than AGPs against pathogen infections, oxidation and inflammation in animal trials [38]. However, the majority of alternative strategies are focused on health; in terms of promotion of growth performance, they yield mostly inconsistent results that are unequal to the effects of AGPs [37]. In addition, the administration route and economic costs indicate that much work is required for large-scale utilization of the listed alternatives for growth promotion. In this context, we focus on a conventional feedstuff, LCFAs, because of their extensive sources, cost effectiveness, safety, oral administration and potential functional roles [39–41]. Based on a systematic, large-scale literature search and meta-analysis, we were able to comprehensively and quantitatively confirm the beneficial effects of LCFAs on pig growth performance. Regression analyses of the basal diet, concentration and saturation (Table 4) together with subgroup analysis of the concentration in finisher pigs (Figs. 2 and 3) further suggested that the benefits were concentration-dependent.

As shown in Table 2, the improved changes in growth performance of the growing and finishing pigs fed different LCFAs were mainly associated with the energy level. Of the 18 studies (26 records) included in our meta-analysis, 13 (20 records) revealed an elevated energy density after LCFA supplementation. Thus, even a lower feed intake is able to meet the caloric requirements of pigs. Moreover, intake of additional LCFAs will improve the digestibility of amino acids by lowering the gastric emptying speed and increasing the time of exposition to proteolytic enzymes [42–44]. As a consequence, a higher ratio of amino acids in feed will participate in meat production, which in turn increases the weight gain and gain efficiency.

Our findings revealed no differences in the effects of saturated and unsaturated LCFAs on growth performance (Table 4). Unsaturated fatty acids (e.g., linoleic acid) are essential in pig feed because of the absence of desaturase enzymes [9]. In pig farming, essential unsaturated fatty acids improve sow fertility and piglet growth [45–47] via their beneficial effects on neural development, immune responses and gut health [39–41]. Nevertheless, for growers and finishers, whose body systems are highly mature, the primary role of fatty acids is to be oxidized and to store and supply energy. Additionally, the regression analysis indicated that the addition of DDGS to a corn-soybean diet did not impair the promoting function of LCFAs, which was in accordance with the review of Stein and Shurson [48].

As shown in Fig. 3, the significant heterogeneity in the ADFI and G:F ratio of the finishers was primarily driven by the high-concentration subgroup. In the sensitivity analyses, we found that Villela et al. [32] (2 records) and Liu et al. [34] (4 records) were the major sources of heterogeneity (data not shown). After excluding the 2 studies (6 records), the overall and high-concentration subgroup heterogeneity in the ADFI became nonsignificant. Additionally, exclusion of the 6 records from the ADFI analysis caused the originally negative effects of LCFA supplementation on the overall group and high-concentration subgroup to become nonsignificant. For the G:F ratio, removing the 6 records decreased the heterogeneity but did not influence the significance of the pooled estimates. The differences in heterogeneity and statistical significance of the pooled estimates were primarily due to the variation in energy density. In the trial of Villela et al. [32], the metabolizable energy of the diet with 5% minimally refined cottonseed oil (3537 kcal/kg) in two phases (55–90 kg and 90–120 kg) was 253 kcal/kg higher than that of the control diet (3284 kcal/kg). Because the gossypol concentration (0.001%) was too low to exert any adverse effects on the growth performance [49], Villela et al. [32] concluded that the energy density was the key reason for the changes in growth performance. To clarify the concentration dependency of the results, we initially set the concentrations to approximately 1% and 5% for the low- and high-concentration groups, respectively (Table 2). The concentration applied in the study of Liu et al. [41] was the highest (6%) among the included studies, and the energy difference between the group with 6% lipid supplementation (3600 kcal/kg) and the control group (3320 kcal/kg) was also considerable (270 kcal/kg). Therefore, a lower ADFI still provided sufficient energy for weight gain and enhanced the gain efficiency, indicating that the promotion of growth performance by LCFAs was related to an increased energy density.

The limitation of our study is the effects of within-group SD estimates on the pooled estimates. Due to the lack of within-group SDs in animal nutrition studies, we had to perform our meta-analysis based on within-group SDs estimated using 8–15% of the mean values. Because SD values impact the 95% CIs and the weight of an individual study, they also affect the pooled estimates and heterogeneity. To improve the validity of our findings, we compared the estimate range with the true values to test its accuracy. With data provided by the Moran group, we found that the true within-group SDs of the ADG and G:F ratio were precisely located within our estimate range. In contrast, the SD for the ADFI was under the lower limit because of the data collection method. Unlike the ADG and G:F ratio, in most cases the ADFI of each commercial pig can be obtained only by dividing the total intake per pen by the number of pigs. This method masks individual variation, causing a lower within-group SD than we would predict. Similar to the SDs of the ADG and G:F ratio, the variation in ADFI should be consistent with the calculated SD range. However, the dominant contributor to the pooled estimates was the mean difference of each study instead of the 95% CI and weight. The random-effects model we used was capable of balancing the differences in individual weights and therefore highlighting the role of mean differences. Taken together, both the accurate SD estimates and the primary role of the mean difference between the treatment and control groups ensure that this meta-analysis conducted on within-group SD estimates is reliable.

## Conclusions

Our results indicate that LCFA supplementation of feed improves the ADG and G:F ratio of both growers and finishers, whereas LCFA supplementation leads to a reduction in the ADFI of finishers. Moreover, for finishers, only a high LCFA concentration (approximately 5%) is capable of enhancing the ADG and G:F ratio and decreasing the ADFI, whereas the basal diet category (corn-soybean vs. DDGS) and the saturation level (saturated vs. unsaturated) have small effects on the ADG, ADFI and G:F ratio of finisher pigs. These findings indicate that the positive effects of LCFA supplementation result from an increased energy density. Further experimental research is required to establish the optimal supplemental LCFA concentration and to explore appropriate sources.

## Data Availability

All data generated or analysed during this study are included in this published article.

## Abbreviations

ADFI: Average daily feed intake
ADG: Average daily gain
AGP: Antimicrobial growth promoter
CI: Confidence interval
CLA: Conjugated linoleic acid
CWG: Choice white grease
DDGS: Distillers’ dried grains with solubles
DHA: Docosahexaenoic acid
G:F ratio: Gain: Feed ratio
SD: Standard deviation
SE: Standard error
WMD: Weighted mean difference
